# Edge–Interior Differences in Plant Diversity and Herbaceous Functional Composition in Tropical Urban Remnant Forests: Associations with Invasive-Herb Dominance

**DOI:** 10.3390/plants15192964

**Published:** 2026-09-29

**Authors:** Fangfei Lu, Rongxiao He, Mingze Xu, Yiying Wu, Yu Zhu

**Affiliations:** School of Tropical Agriculture and Forestry, Hainan University, Haikou 570228, China; lufangfei@hainanu.edu.cn (F.L.);

**Keywords:** urban remnant forests, edge effects, invasive herbs, species origin, community-weighted means

## Abstract

Higher plant richness at forest edges does not by itself reveal whether native diversity is retained. We surveyed 196 plots in 28 tropical urban remnant forest patches in Haikou, China, comparing edge and interior vegetation. Herb richness and Shannon diversity were higher at edges, whereas adult-tree richness was lower. The total-herb-richness increase (1.94 species plot^−1^) was concentrated in invasive herbs (2.26 species plot^−1^); neither native-herb nor alien non-invasive-herb richness differed after FDR adjustment, whereas invasive-herb projected area increased. Edges also differed in projected-area-weighted functional composition, but richness-standardized functional diversity did not differ. Balanced Hill analyses corroborated higher local edge diversity at q = 0, 1, and 2, but this pattern did not translate into a parallel study-wide pooled gain in typical or dominant herb diversity. Edge communities were less dissimilar among patches under Bray–Curtis and Jaccard indices, and excluding widespread invasive herbs weakened these contrasts. Higher local herb diversity at forest edges therefore reflected changes in species origin, dominance, and among-patch composition rather than a uniform increase across species-origin groups, highlighting urban and peri-urban forest edges as priorities for invasive-herb surveillance and timely control.

## 1. Introduction

Urban remnant forests are natural or semi-natural forest patches retained within landscapes transformed by urbanization and can serve as important reservoirs of native biodiversity [1,2,3]. Typically occurring as relatively small and isolated patches, their plant communities are shaped not only by patch attributes but also by the composition and configuration of the surrounding urban matrix [4,5,6]. Habitat fragmentation reduces forest area and increases the proportion of habitat exposed to patch boundaries, thereby increasing the potential influence of edge-related environmental and biotic changes [7,8].

Edge effects are an important consequence of habitat fragmentation through which the surrounding matrix alters abiotic conditions, species diversity, and community composition within remnant forest patches [8,9,10]. The magnitude and direction of plant responses vary with edge type, matrix contrast, patch configuration, edge age, and species ecological characteristics [11,12,13]. In urban remnant forests, edge-related differences have been reported in woody plant diversity, species composition, functional traits, and stand structure, although the direction and strength of these responses differ among cities and landscape contexts [14,15,16]. Recent syntheses indicate that the effects of patch edges on plant diversity are context dependent across vegetation types and spatial scales [17,18]. Thus, edge effects cannot be characterized as a uniform increase or decline in plant diversity.

Previous studies have shown that edge responses vary among vegetation layers and woody ontogenetic stages. Adult trees represent established community structure, whereas saplings and seedlings reflect regeneration; responses in shrubs may differ further because they occupy a lower vegetation layer [16,19,20]. Accordingly, evidence from a single vegetation layer or developmental stage cannot characterize edge–interior responses across all vegetation layers.

Despite this progress, research on plant responses in urban remnant forests has predominantly focused on woody vegetation, leaving herbaceous communities comparatively understudied. Herbaceous plants account for a substantial share of forest vascular-plant diversity and contribute to nutrient cycling, litter decomposition, soil processes, and tree regeneration [21,22]. Studies of forest understory communities also show that herbaceous diversity responds to stand structure, topography, and local environmental conditions [23,24]. Because herbs occupy the near-ground environment and generally have shorter life cycles and faster turnover than woody plants, they can respond rapidly to changes in canopy openness, microclimate, drought exposure, and disturbance [25,26,27,28]. Forest edges are also exposed to propagule inputs from surrounding urban land uses, and alien plants frequently differ from native plants in establishment patterns and functional traits [29,30,31]. Invasive alien plants can alter resident communities and ecosystem properties [32], whereas the spread of widespread alien species may reduce compositional differentiation among local floras and contribute to floristic homogenization [33]. Consequently, studies restricted to woody plants may overlook edge-associated changes in herbaceous diversity, species origin, dominance, functional composition, and among-patch differentiation.

However, it remains unclear whether edge–interior differences in herbaceous diversity are accompanied by shifts in species origin, invasive-herb dominance, functional composition, and compositional differentiation among urban forest remnants. We expected edge–interior responses to differ among vegetation layers, such that a single whole-community diversity summary may not reveal whether an edge–interior pattern arises from woody vegetation, the herbaceous layer, or both. We further expected that any edge-associated increase in herbaceous diversity would depend on species origin and dominance and might differ between local diversity and diversity at the study-wide pooled scale. In this study, we used a within-patch edge–interior sampling design across 28 tropical urban remnant forest patches in Haikou, China. Specifically, we aimed to: (1) quantify edge–interior differences in taxonomic diversity across vegetation layers and assess the contributions of herbaceous species-origin groups to herb-layer responses; (2) assess whether invasive-herb dominance was associated with differences in projected-area-weighted functional composition and richness-standardized functional diversity of herbaceous communities; and (3) characterize edge–interior differences in herbaceous diversity across local alpha diversity, study-wide pooled gamma diversity across the sampled forest-patch network, and among-patch compositional differentiation.

## 2. Materials and Methods

### 2.1. Study Area and Selection of Urban Remnant Forest Patches

The study was conducted in Haikou, Hainan Province, southern China, within 19.9–20.1° N and 110.2–110.4° E. Haikou has a tropical maritime monsoon climate, with a mean annual temperature of approximately 24.4 °C and mean annual precipitation of approximately 1696.6 mm. The regional natural vegetation is tropical monsoon forest, although long-term urban expansion and land-use change have fragmented part of the original forest cover into remnants embedded within the urban landscape.

Candidate forest patches were screened using the available maps spanning 1980–2025 from the China Land Use and Land Cover Change dataset (CNLUCC; 30-m spatial resolution), together with the 2023 GLC_FCS10 land-cover map [34,35]. The historical land-use maps were used to identify candidate areas repeatedly mapped as forest across the available observation years, whereas GLC_FCS10 was used to delineate contemporary forest cover and patch boundaries. Candidate patches were subsequently inspected in the field, and plantations, orchards, nurseries, and stands subjected to recent intensive human modification were excluded. A total of 28 urban remnant forest patches, ranging from 6.47 to 31.44 ha, were retained for vegetation sampling (Figure 1).

### 2.2. Edge–Interior Sampling and Vegetation Survey

Four edge plots and three interior plots, each measuring 20 m × 20 m, were established in each patch, resulting in 196 plots in total. The placement of edge plots followed previous forest edge-effect studies and sampling designs used in urban remnant forests [13,19,36]. The four edge plots were located on the eastern, southern, western, and northern sides of each patch, with plot centers approximately 25 m from the forest boundary. The three interior plots were placed within the interior portion of each patch, as far from the nearest forest boundary as patch size and geometry allowed, while avoiding roads, building boundaries, and other evident sources of intensive anthropogenic disturbance. For analyses conducted at the patch × sampling-position level, community metrics were averaged separately across the four edge plots and the three interior plots within each patch. A common 20 m × 20 m sampling unit was used for all vegetation layers to characterize co-occurring vegetation within the same spatial footprint and to permit paired edge–interior comparisons. Hereafter, sampling position refers to edge or interior. Each plot was surveyed between November 2025 and May 2026.

All vascular plants were surveyed in four vegetation layers: adult trees, saplings, shrubs, and herbs. Tree individuals were classified as adult trees (diameter at breast height [DBH] ≥ 3 cm) or saplings (DBH < 3 cm) following Fang et al. [37]. For adult trees, species identity, individual abundance, and DBH were recorded. For saplings, shrubs (including woody vines), and herbs (including non-woody vines), species identity and species-level horizontal projected area (m^2^) were recorded within each 400 m^2^ plot. For each species, projected area was estimated as the horizontal ground area occupied by its above-ground vegetation within a plot and recorded in m^2^ rather than assigned to a cover class. Estimates were made by the same survey team using a consistent protocol. Because species could overlap vertically, the summed projected areas of species within a plot could exceed 400 m^2^.

### 2.3. Species Origin and Plant Functional Traits

The biogeographic origin and invasion status of herbaceous species were assigned with reference to the Catalogue of Alien Plants in China and recent national inventories of alien and invasive plants [38,39]. Herbaceous species were classified into three mutually exclusive groups: native herbs, alien non-invasive herbs, and invasive alien herbs (hereafter invasive herbs; Table S1). The three groups were summarized and evaluated separately. Because alien non-invasive herbs were relatively infrequent (11 species), native and alien non-invasive herbs were additionally combined only for a prespecified pooled non-invasive comparison with invasive herbs and for the corresponding functional-composition sensitivity analysis. Separate native-herb and alien non-invasive-herb richness models are reported in Table S2. Absolute invasive-herb projected area was the summed projected area of invasive herbs in each plot, and relative invasive-herb projected-area share was absolute invasive-herb projected area divided by total herbaceous projected area. All plots contained at least one herbaceous species; therefore, total herbaceous projected area was greater than zero in every plot.

Five functional traits representing plant size, leaf resource-use strategy, and regeneration were considered: maximum plant height, specific leaf area (SLA), individual leaf area, leaf dry matter content (LDMC), and thousand-seed mass. Because the herbaceous layer included ferns, thousand-seed mass was applicable only to seed plants, whereas the other four traits were applicable across all recorded herbaceous species. Trait definitions and units followed standardized measurement protocols [40]. Trait values were compiled primarily from the TRY Plant Trait Database [41] and supplemented with floristic sources and published trait records. When multiple records were available for a species, trait values were averaged after unit harmonization. At the species level, maximum plant height, individual leaf area, and thousand-seed mass were log_10_-transformed, whereas SLA and LDMC were retained on their original scales. Each trait was subsequently z-standardized across herbaceous species with available values.

Community-weighted means (CWMs) were calculated for each plot using relative projected area as the abundance weight. For each trait, species projected areas were re-normalized among species for which the trait was applicable and available before the CWM was calculated. CWMs were calculated for the complete herbaceous community and recalculated after invasive herbs were excluded to assess the sensitivity of edge–interior differences in functional composition to their inclusion.

### 2.4. Taxonomic Diversity, Functional Diversity, and Community Composition

Species richness and Shannon diversity were calculated separately for adult trees, saplings, shrubs, and herbs at the plot level. For adult trees, species importance values were calculated as the mean of relative abundance and relative basal area and were used as species weights for calculating Shannon diversity. For saplings, shrubs, and herbs, relative projected area was used as the species weight. Edge–interior comparisons were conducted separately within each vegetation layer; diversity values were not compared directly among vegetation layers. Surveyed plot–layer combinations with no recorded species were assigned richness and Shannon diversity values of zero in the primary analyses. Because plots without saplings were relatively frequent, sapling Shannon diversity was additionally evaluated in a sensitivity analysis restricted to plots in which saplings were present. All 196 plots were retained as individual observations in the taxonomic-diversity and all-herb CWM analyses. In the pooled non-invasive herb CWM sensitivity analysis, three plots contained no native or alien non-invasive herb species after invasive herbs were excluded. These plots were omitted because the corresponding CWMs were undefined, resulting in 193 plots from all 28 patches. Edge and interior values were averaged only for analyses explicitly conducted for each patch and sampling position. Model specifications for the taxonomic-diversity, species-origin, and invasive-herb-dominance analyses are reported in Table S2.

Multidimensional functional diversity was evaluated for each patch and sampling position using four vegetative traits that were applicable to all 126 recorded herbaceous species: log_10_-transformed maximum plant height, SLA, log_10_-transformed individual leaf area, and LDMC. Thousand-seed mass was retained as a regeneration-related trait in the single-trait CWM analyses. Species projected area was averaged across plots belonging to the same sampling position within each patch, and functional richness (FRic), functional evenness (FEve), functional dispersion (FDis), functional divergence (FDiv), and Rao’s quadratic entropy (Rao’s Q) were calculated using the FD package [42,43,44,45]. These indices quantify, respectively, trait-space volume, the regularity of abundance distribution in trait space, abundance-weighted dispersion from the community centroid, abundance-weighted divergence toward trait-space extremes, and abundance-weighted mean pairwise trait dissimilarity. Because functional-diversity indices may covary with species richness, standardized effect sizes were estimated using a richness-constrained null model [46]. For each patch × sampling-position community, species richness was held constant, species were drawn without replacement from a common study-wide species pool comprising all 126 recorded herbaceous species, and the observed positive projected-area vector was retained but randomly reassigned among the sampled species. This null model controlled species richness but did not constrain species occurrence frequencies. The procedure was repeated 999 times, and standardized effect sizes were calculated as the difference between the observed value and the mean of the null distribution divided by its standard deviation. FRic and FDiv could not be estimated for four interior communities because their species richness was insufficient relative to the four-dimensional trait space; mixed-effects analyses retained all estimable communities and included patch identity as a random effect. Trait coverage, CWM results, and richness-standardized functional-diversity results are reported in Table S3.

Differences in herbaceous community composition between edge and interior plots were quantified using Bray–Curtis dissimilarities calculated from untransformed species projected-area data. Permutational multivariate analysis of variance (PERMANOVA) was implemented in the vegan package to test for compositional differences between sampling positions [47]. Because PERMANOVA can be affected by differences in within-group multivariate dispersion, homogeneity of dispersion was assessed using PERMDISP, based on distances to group centroids [48].

To assess compositional differentiation among patches, species projected area was first averaged separately across edge and interior plots within each patch. Pairwise Bray–Curtis and Jaccard dissimilarities were then calculated among edge communities and among interior communities for all herbs, pooled non-invasive herbs, and invasive herbs. Bray–Curtis dissimilarity retains relative-abundance structure, whereas Jaccard dissimilarity is based on species incidence and therefore represents differences in species membership independently of abundance. The analyses were repeated for the full herbaceous community after excluding the three and five invasive herbs with the highest frequencies across the 196 plots to evaluate the contribution of widespread invasive herbs to the observed among-patch patterns.

### 2.5. Hill-Number and Exploratory Patch-Attribute Analyses

To characterize herbaceous diversity across orders of sensitivity to species abundance and across spatial scales, we calculated Hill diversity at q = 0 (species richness), q = 1 (the exponential of Shannon entropy), and q = 2 (the inverse Simpson concentration). To balance the unequal number of edge and interior plots within each patch, all four possible selections of three of the four edge plots were averaged before calculating patch-level alpha, gamma, and multiplicative beta diversity. A complementary study-wide pooled comparison used 4999 balanced selections, each retaining three edge plots per patch, and compared the resulting 84 edge plots with the 84 interior plots. The study-wide pooled selection ranges describe sensitivity to balanced edge-plot selection rather than confidence intervals for patch sampling. Full procedures and estimates are reported in Table S4.

We also explored whether balanced patch-level herb-layer edge effects were associated with patch area, core-area index at 50 m (CAI50), or isolation distance. These exploratory associations were evaluated using Spearman correlations with false-discovery-rate correction and are reported in Table S5.

### 2.6. Statistical Analysis

All analyses were conducted in R version 4.6.0 [49]. Sampling position (interior or edge) was included as a fixed effect, and patch identity was included as a random effect to account for the non-independence of plots within patches. Species-richness responses were analyzed using generalized linear mixed-effects models fitted with glmmTMB [50]. Response-specific count distributions and random-effects structures were selected based on convergence, Akaike’s information criterion (AIC), and simulation-based residual diagnostics using 1000 simulations in DHARMa. When competing models differed by ΔAIC ≤ 2 and showed similarly acceptable residual diagnostics, the simpler distribution or random-effects structure was retained. Random-slope structures were simplified to random-intercept structures when they produced singular, unstable, or non-convergent fits. Shannon diversity and standardized CWM values were analyzed using Gaussian linear mixed-effects models fitted with lme4 [51].

Absolute invasive-herb projected area was analyzed using a Tweedie generalized linear mixed-effects model with a log link, and relative invasive-herb projected-area share was analyzed using an ordered-beta mixed-effects model with a logit link; both models included a random intercept for patch. Estimated marginal means and Edge–Interior contrasts were obtained on the response scale using emmeans, and effect estimates are reported with unadjusted 95% confidence intervals (CIs). The Benjamini–Hochberg procedure controlled the false discovery rate (FDR) [52] separately within five a priori response families: taxonomic diversity, herbaceous species origin, invasive-herb dominance, all-herb CWMs, and functional-diversity SESs. Targeted sensitivity analyses were adjusted within their respective analysis sets.

For plot-level community composition, Bray–Curtis dissimilarities were calculated from untransformed species projected-area data. PERMANOVA was fitted with sampling position as the predictor, and 9999 permutations were restricted within patches. PERMDISP used distances to group centroids, 9999 patch-restricted permutations, and an unequal-sample-size bias adjustment. Among-patch differentiation was quantified as mean pairwise edge–edge dissimilarity minus mean pairwise interior–interior dissimilarity. Its 95% CI was estimated from 4999 paired patch-level bootstrap resamples. In each bootstrap iteration, the 28 patches were resampled with replacement as paired edge–interior units, and distances between repeated copies of the same original patch were excluded. For the permutation tests, edge and interior labels were independently exchanged within each patch. Two-sided *p* values were calculated as (*b* + 1)/(*B* + 1), where b was the number of permuted absolute contrasts at least as large as the observed absolute contrast and B = 9999. The contribution of widespread invasive herbs was evaluated by repeating the analysis after excluding the three and five most frequent invasive herbs.

## 3. Results

### 3.1. Taxonomic Diversity Across Vegetation Layers

Edge–interior differences varied among vegetation layers (Figure 2; Table S2, Panel A). Herb richness was 1.94 species plot^−1^ higher at edges (95% CI: 1.24–2.64; FDR-adjusted *p* < 0.001), and herb Shannon diversity was 0.402 higher (95% CI: 0.220–0.584; FDR-adjusted *p* < 0.001). Adult-tree richness was lower at edges, whereas sapling and shrub responses were negative or sensitive to analytical specification (Table S2). These layer-specific results show that the components underlying edge–interior patterns differed among vegetation layers.

Exploratory associations between patch attributes and herb-layer edge effects were not supported after FDR adjustment (Table S5).

### 3.2. Species Origin and Dominance by Invasive Herbs in the Herbaceous Layer

A total of 334 vascular plant species were recorded across the four vegetation layers. The herbaceous layer contained 126 species: 91 native, 11 alien non-invasive, and 24 invasive species (Table S1). Observed mean herb richness was 5.26 species plot^−1^ in interior plots and 7.23 species plot^−1^ at edges (Figure 3A).

The edge-associated increase in total herb richness was concentrated in invasive herbs (edge − interior = 2.26 species plot^−1^, 95% CI = 1.89–2.63, FDR-adjusted *p* < 0.001), whereas pooled non-invasive-herb richness did not differ between positions. Separate models detected no edge–interior difference in either native-herb richness or alien non-invasive-herb richness after FDR adjustment (Figure 3B; Table S2, Panel B). Thus, the observed edge-associated increase in total herb richness cannot be interpreted as an increase in native-herb richness.

Invasive herbs were also more dominant at edges (Figure 3C). Absolute invasive-herb projected area was 57.87 m^2^ higher at edges (95% CI: 44.80–70.94; FDR-adjusted *p* < 0.001), and relative invasive-herb projected-area share was 36.86 percentage points higher (95% CI: 31.28–42.44; FDR-adjusted *p* < 0.001).

### 3.3. Hill-Number Diversity Across Local and Study-Wide Pooled Scales

Balanced Hill-number analyses showed that herbaceous alpha diversity was higher at edges at q = 0, q = 1, and q = 2, indicating that the edge pattern was evident not only for rare species but also when common and dominant species received greater weight. At the study-wide pooled scale, edge communities had higher gamma diversity at q = 0, whereas interior communities had higher study-wide pooled gamma diversity at q = 1 and q = 2. Multiplicative beta diversity was lower at edges across all three orders, indicating lower among-patch differentiation of edge herb communities. Thus, the increase in local edge diversity did not translate into a parallel study-wide pooled gain in typical or dominant herb diversity (Figure 4; Table S4). The intervals in Figure 4 describe sensitivity to balanced edge-plot selection rather than confidence intervals for patch sampling.

### 3.4. Functional Composition and Functional Diversity of Herbaceous Communities

Functional composition differed between sampling positions (Figure 5; Table S3). Relative to interior plots, edge communities had lower CWMs of plant height, leaf area, and seed mass and a higher CWM of SLA, whereas LDMC did not differ after FDR adjustment.

After invasive herbs were excluded, none of the five CWM traits differed between edge and interior communities after FDR adjustment (Table S3). The complete-community CWM contrasts were therefore not retained in the pooled non-invasive sensitivity analysis.

Richness-standardized functional diversity did not differ between edge and interior communities for FRic, FEve, FDis, FDiv, or Rao’s Q after FDR adjustment (Table S3).

### 3.5. Among-Patch Compositional Dissimilarity and Widespread Invasive Herbs

PERMANOVA detected edge–interior differences in Bray–Curtis community structure (R^2^ = 0.061, *p* < 0.001). However, patch-restricted PERMDISP also detected different multivariate dispersion between edge and interior plots (F = 43.79, *p* < 0.001); mean distance to the group centroid was 0.638 for interior plots and 0.561 for edge plots. Thus, the PERMANOVA result reflects edge–interior separation together with differences in multivariate dispersion.

Consistent with the lower multiplicative Hill beta diversity at edges, Bray–Curtis and Jaccard dissimilarities were also lower among edge than among interior herb communities (Figure 6). For all herbs, the edge–edge minus interior–interior contrast was −0.169 for Bray–Curtis (95% CI: −0.216 to −0.118; permutation *p* < 0.001) and −0.094 for Jaccard (95% CI: −0.125 to −0.063; *p* < 0.001). Corresponding contrasts were smaller for pooled non-invasive herbs and largest for invasive herbs. Thus, edge communities were more similar to one another across patches than were interior communities at the time of sampling.

The three invasive herbs with the highest frequencies across the 196 plots were *Chromolaena odorata*, *Mikania micrantha*, and *Bidens alba*, recorded in 97, 78, and 62 plots, respectively. Excluding the three or five most widespread invasive herbs shifted the edge–edge versus interior–interior dissimilarity contrasts toward zero; after the five-species exclusion, the Jaccard contrast was no longer supported (Figure S3). Widespread invasive herbs therefore contributed substantially to the lower among-patch differentiation of edge communities.

## 4. Discussion

Our results show that evaluating edge effects in tropical urban remnant forests requires more than total plant richness. The clearest positive response occurred in the herbaceous layer, where higher local richness was concentrated in invasive herbs and accompanied by substantially greater invasive-herb projected area. These changes in species origin and dominance coincided with projected-area-weighted shifts in functional composition and lower among-patch compositional differentiation, particularly among invasive-herb assemblages. Together, these results show that apparent increases in local diversity at urban forest edges can mask changes in species origin, dominance, and community structure.

### 4.1. Edge Effects Across Vegetation Layers

Edge–interior differences varied among vegetation layers. Herb richness and Shannon diversity were consistently higher at edges, whereas adult-tree richness was lower; sapling and shrub responses were less consistent across analytical specifications. Similar contrasts have been reported in urban remnant forests, where adult trees, saplings or seedlings, and shrubs respond differently to edge exposure and surrounding urbanization, and where changes in species composition or functional traits do not always coincide with changes in species diversity [14,15,16,19]. By surveying all four vegetation layers in the same patches, our study shows that positive diversity responses were concentrated in the herbaceous layer. The lower adult-tree richness at edges indicates that the established woody layer was less diverse in the same patches in which herbaceous richness and invasive-herb dominance increased.

### 4.2. Species Origin and the Interpretation of Herbaceous Diversity

Partitioning herbaceous richness by species origin substantially changed the interpretation of the edge response. The model-estimated increase in invasive-herb richness at forest edges (2.26 species per plot) closely corresponded to the increase in total herb richness (1.94 species per plot), whereas neither native-herb nor alien non-invasive-herb richness showed an edge–interior difference after FDR adjustment. Absolute invasive-herb projected area and relative invasive-herb projected area share were also substantially higher at edges. Thus, the edge response involved both higher invasive-herb richness and greater dominance by invasive herbs, rather than a general increase across all species-origin groups.

Studies of urban subtropical and tropical forest remnants show that propagule input from surrounding human-modified land uses and edge-associated disturbance can favor plant invasion [29,53,54]. Complementary evidence from natural and temperate forests indicates that local disturbance and habitat degradation can intensify understorey invasions [55,56]. Studies of tropical rainforest remnants further show that exotic species from surrounding human-modified habitats do not establish equally across forest conditions, because establishment in forest understories is filtered by disturbance history and local habitat conditions [57]. In Haikou, the edge-associated increase in invasive herbs is consistent with these documented processes, but the present study cannot distinguish their relative contributions.

Accordingly, total herb richness alone cannot distinguish native-herb retention from increased representation and dominance of invasive herbs; species origin and relative abundance are needed to interpret edge-associated richness patterns. Invasive alien plants can alter resident communities and ecosystem properties, and alien plant assemblages can differ in both composition and function from native floras [32,58]. At broader spatial scales, the introduction of exotic species has contributed to floristic homogenization in China [33].

### 4.3. Dominance by Invasive Herbs, Functional Composition, and Functional Diversity

Edge communities differed in projected-area-weighted functional composition, with higher SLA and lower CWMs of plant height, leaf area, and seed mass, whereas richness-standardized functional diversity did not differ. Thus, the edge pattern involved a shift in the trait composition of dominant herbaceous vegetation rather than a detectable expansion or contraction of multivariate trait space. After invasive herbs were excluded, the CWM contrasts were no longer supported, indicating that their inclusion contributed to the observed community-level functional pattern. Because CWMs summarize abundance-weighted community composition, these results do not imply a uniform trait profile for individual invasive and non-invasive species. Trait filtering during exotic plant invasion has also been documented in tropical rainforest remnants, but the traits associated with establishment vary among disturbance gradients and forest contexts [57].

### 4.4. Local Diversity and Among-Patch Compositional Differentiation

Edge–interior herbaceous diversity patterns differed across local, study-wide pooled, and among-patch dimensions. Balanced Hill-number analyses showed that the higher local herb diversity at edges was evident at q = 0, q = 1, and q = 2. At the study-wide pooled scale, however, higher edge gamma diversity was confined to q = 0; typical and dominant herb diversity was higher in interior communities at q = 1 and q = 2. This contrast shows that an edge-associated increase in local richness did not represent a parallel study-wide pooled gain in typical or dominant herb diversity (Table S4; Figure 4).

Edge communities were also less dissimilar among patches than interior communities under both Bray–Curtis and Jaccard indices, with the greatest difference observed for invasive herbs. This combination of higher local richness and lower among-patch dissimilarity indicates that edge-associated increases in alpha diversity can coexist with greater spatial similarity among remnant forest patches. Because multivariate dispersion differed between positions, the inference about among-patch differentiation is based primarily on the direct edge–edge and interior–interior dissimilarity comparisons, rather than on PERMANOVA alone.

Excluding the three or five most widespread invasive herbs shifted the dissimilarity contrasts toward zero, and the Jaccard confidence interval included zero after five species were excluded (Figure S3). This result indicates that a small number of widespread invasive herbs contributed substantially to the lower among-patch differentiation at edges. Taken together, the increase in local herb richness at forest edges was accompanied by greater dominance by invasive herbs and lower among-patch compositional differentiation. These results identify urban and peri-urban forest edges as priority locations for surveillance and timely control of widespread invasive herbs.

## 5. Conclusions

Edge effects in Haikou’s tropical urban remnant forests were concentrated in the herbaceous layer and differed among complementary dimensions of community structure. Higher herb richness and Shannon diversity at edges were associated primarily with greater invasive-herb richness and dominance, whereas neither native-herb nor alien non-invasive-herb richness differed between positions after FDR adjustment. These differences coincided with projected-area-weighted shifts in functional composition, but not richness-standardized functional diversity. Although edge plots supported higher local herbaceous alpha diversity, this pattern did not represent a parallel study-wide pooled gain in typical or dominant herb diversity and was accompanied by lower among-patch differentiation of edge communities. Widespread invasive herbs contributed to the lower among-patch differentiation pattern. In practice, urban and peri-urban forest edges are practical priorities for surveillance and timely control of widespread invasive herbs. Management may also seek to limit avoidable propagule inputs from adjacent land uses and maintain interior forest conditions.

## Figures and Tables

**Figure 1:**
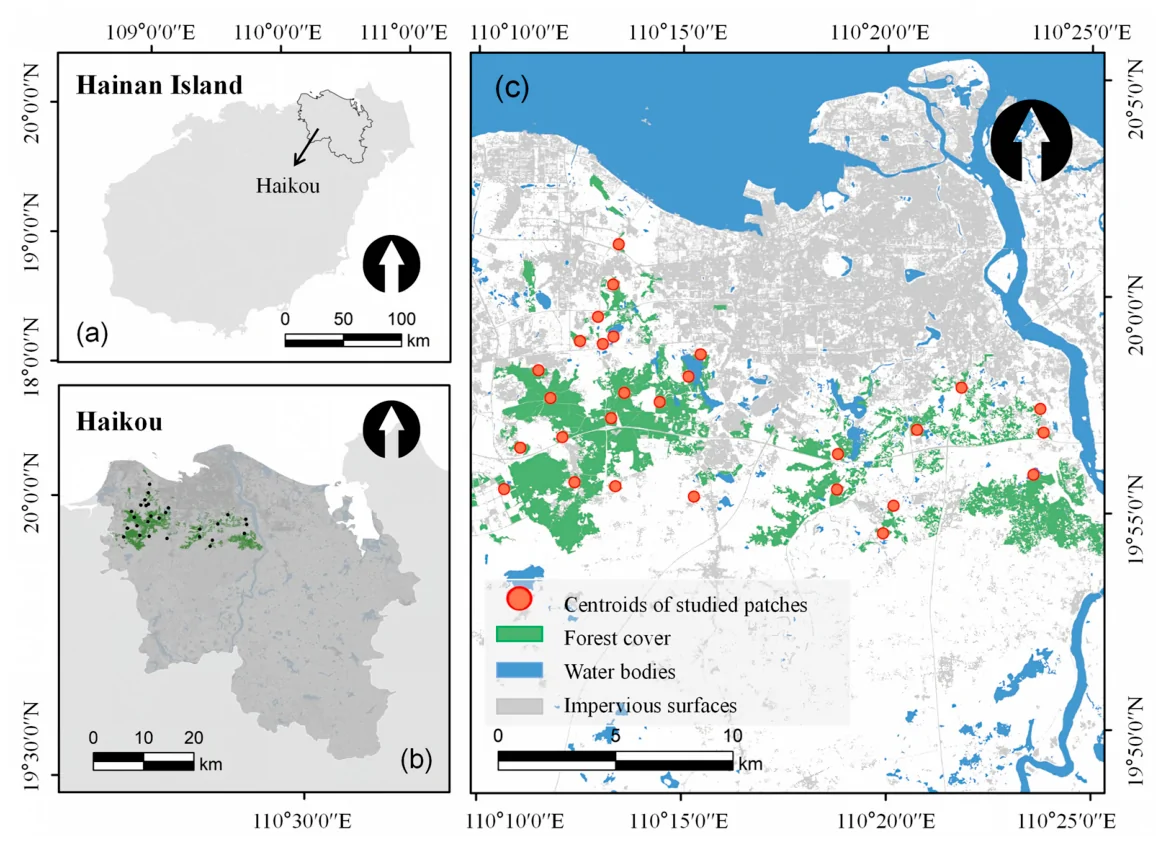
Location of the study area and spatial distribution of the 28 sampled urban remnant forest patches in Haikou, Hainan Island, China. (**a**) Geographic location of Haikou on Hainan Island; (**b**) distribution of the sampled forest patches within Haikou and the extent of the area enlarged in panel (**c**); and (**c**) enlarged view showing the spatial distribution of the sampled patch centroids and forest cover.The arrows in all map panels indicate north.

**Figure 2:**
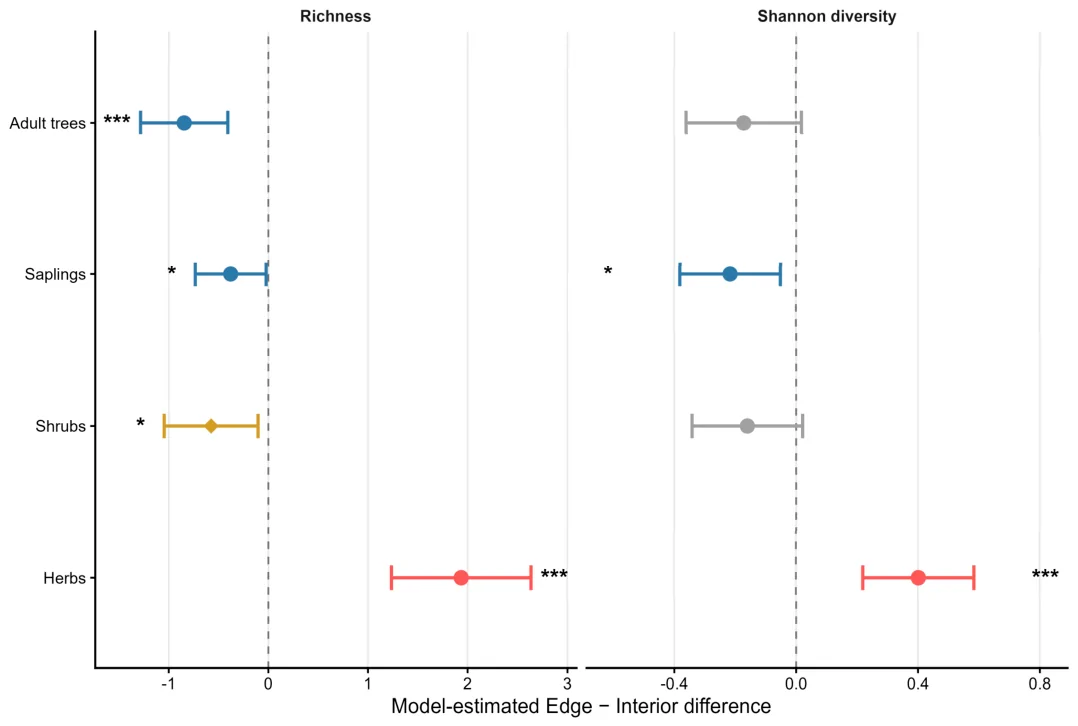
Edge–interior differences in species richness and Shannon diversity across vegetation layers. Effect sizes are model-estimated Edge–Interior differences; positive values indicate higher values at edges, whereas negative values indicate higher values in interior plots. Points and horizontal lines represent model estimates and unadjusted 95% confidence intervals (CIs), respectively. Red and blue circles indicate positive and negative contrasts supported after false-discovery-rate (FDR) adjustment, respectively, whereas gray circles indicate unsupported contrasts. The golden diamond denotes the shrub-richness estimate, for which statistical support was sensitive to model specification. Asterisks indicate FDR-adjusted significance: * *p* < 0.05 and *** *p* < 0.001.

**Figure 3:**
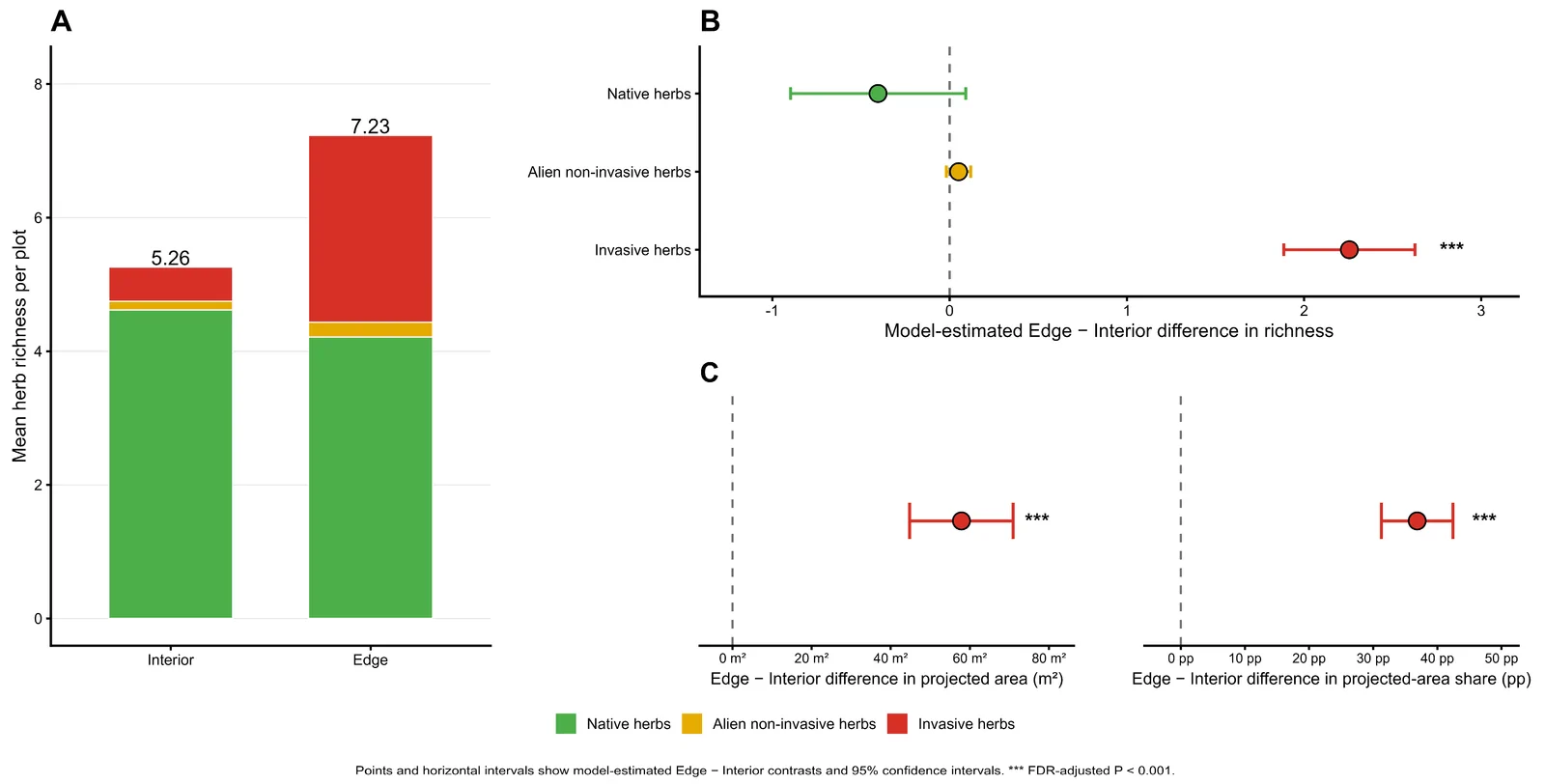
Herbaceous species origin and invasive dominance at forest edges. (**A**) Mean plot-level herb richness partitioned into native, alien non-invasive, and invasive herbs; values above bars indicate total mean herb richness. (**B**) Model-estimated edge—interior differences in richness for native, alien non-invasive, and invasive herbs. (**C**) Model-estimated edge—interior differences in absolute invasive-herb projected area (m^2^) and relative invasive-herb projected-area share (percentage points, pp). Points and horizontal intervals show estimated contrasts and 95% confidence intervals. Asterisks indicate FDR-adjusted significance (*** *p* < 0.001).

**Figure 4:**
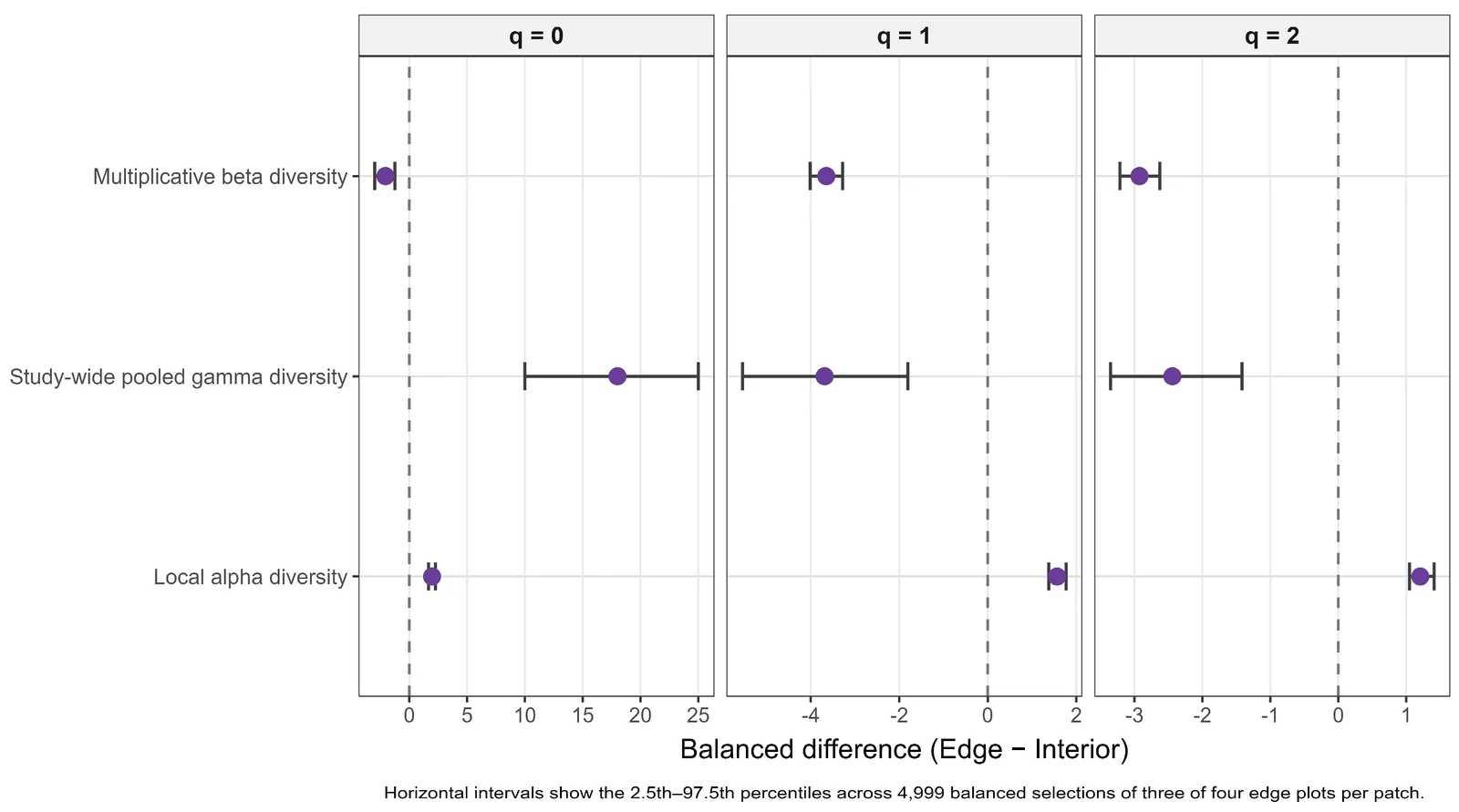
Balanced Hill-number partition of herbaceous communities across the sampled forest-patch network. Points show Edge–Interior differences in local alpha diversity, study-wide pooled gamma diversity, and multiplicative beta diversity at q = 0, 1, and 2. Horizontal intervals show the 2.5th–97.5th percentiles across 4999 balanced selections of three of four edge plots per patch. Positive values indicate higher diversity at edges.

**Figure 5:**
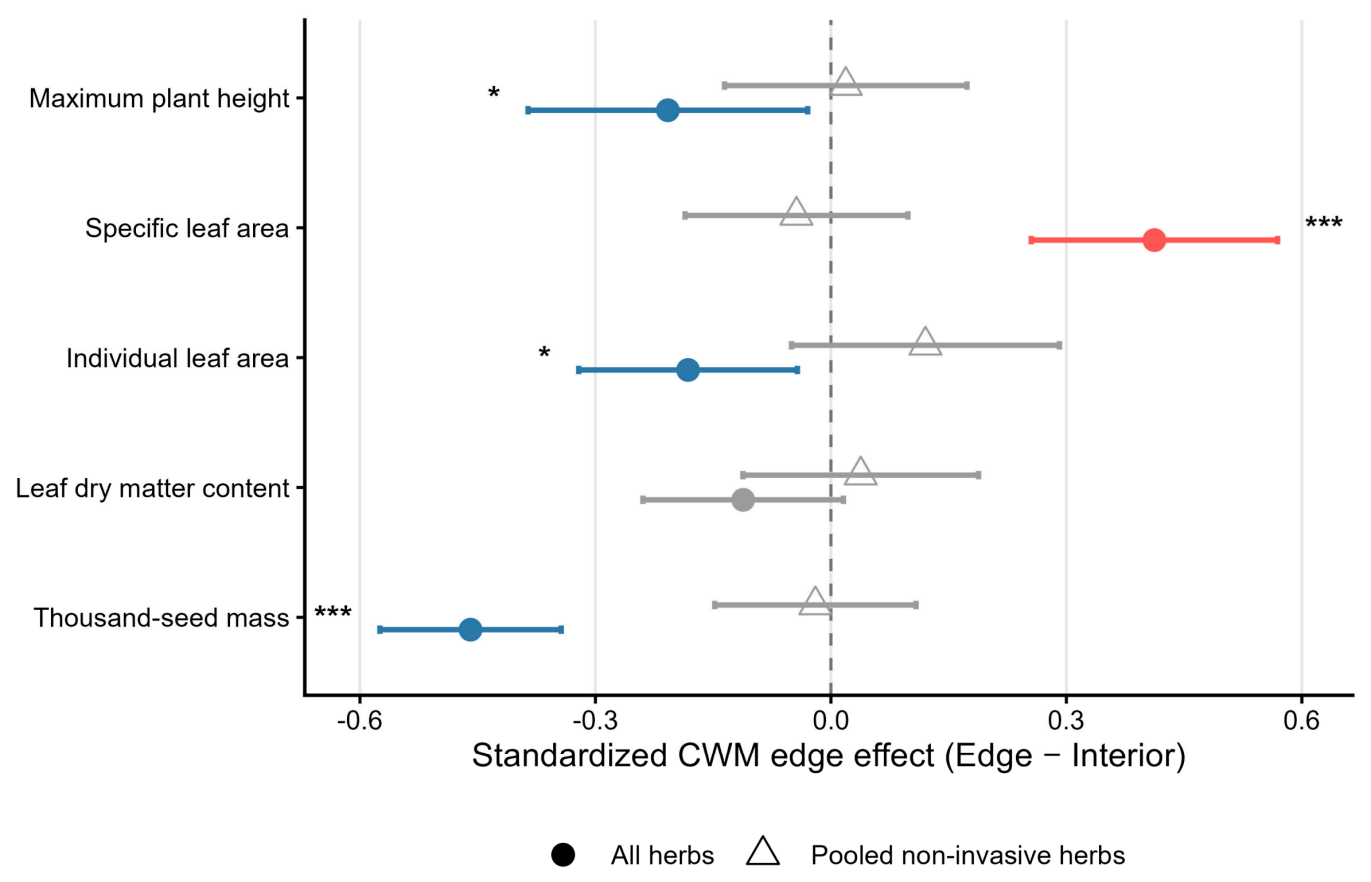
Edge–interior differences in projected-area-weighted functional composition of herbaceous communities. Effect sizes are standardized model-estimated Edge–Interior differences in community-weighted means (CWMs). Solid circles show all-herb communities, whereas open triangles show the pooled non-invasive-herb sensitivity analysis after invasive herbs were excluded. Positive values indicate higher CWM values at edges. Points and horizontal lines show estimates and unadjusted 95% CIs. Colors indicate contrasts supported after FDR adjustment: red, positive; blue, negative; gray, unsupported. Plant height, leaf area, and seed mass were log_10_-transformed prior to standardization. Note: * means *p*-value < 0.5, *** means *p*-value < 0.001.

**Figure 6:**
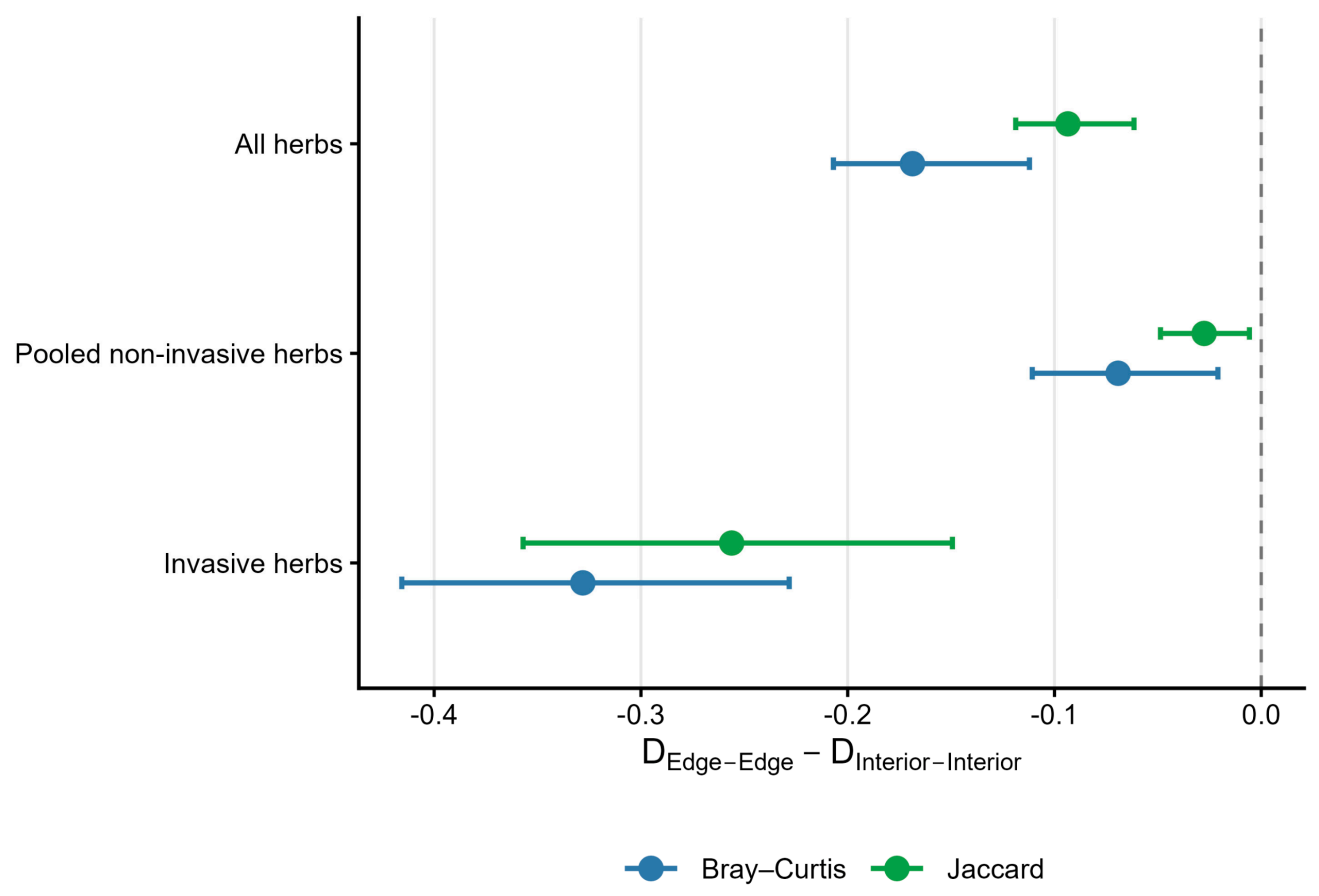
Differences in among-patch compositional dissimilarity between edge and interior herbaceous communities. The horizontal axis shows mean edge–edge dissimilarity minus mean interior–interior dissimilarity for all herbs, pooled non-invasive herbs, and invasive herbs. Negative values indicate lower among-patch differentiation at edges. Blue and green symbols represent Bray–Curtis and Jaccard dissimilarities, respectively; Bray–Curtis represents projected-area-weighted dissimilarity, whereas Jaccard represents incidence-based dissimilarity. Points show observed differences, and horizontal lines show unadjusted 95% CIs from 4999 paired patch-level bootstrap resamples.

## Data Availability

The quantitative vegetation records, plot and patch metadata, species-origin metadata, and layer-specific quantitative community matrices supporting the findings of this study are provided in Supplementary Dataset S1.

## Supplementary Materials

The following supporting information can be downloaded at: https://www.mdpi.com/article/10.3390/plants15192964/s1, Table S1: Herbaceous plant checklist and species origin; Table S2: Mixed-effects model results for taxonomic diversity, herbaceous species origin, and invasive-herb dominance; Table S3: Community-weighted mean traits, trait coverage, and richness-standardized functional diversity; Table S4: Balanced Hill-number analyses across vegetation layers and spatial scales; Table S5: Exploratory associations between patch attributes and balanced herb-layer edge effects; Figure S1: Plot-level Hill diversity across vegetation layers; Figure S2: Balanced patch-level Hill-diversity effects across vegetation layers; Figure S3: Sensitivity of among-patch compositional differentiation to the exclusion of widespread invasive herbs; Dataset S1: Quantitative vegetation records, plot and patch metadata, species-origin metadata, and layer-specific quantitative community matrices.
