# Supplementary material for: Edge–Interior Differences in Plant Diversity and Herbaceous Functional Composition in Tropical Urban Remnant Forests: Associations with Invasive-Herb Dominance
Source: Plants (Basel). 2026 Sep 29;15(19):2964. doi: 10.3390/plants15192964 (PMC13667045; doi:10.3390/plants15192964)
Supplement: Supplementary file 1 [file plants-15-02964-s001.zip › Supplementary_Materials.pdf]

Supplementary Materials

Mixed-effects model results, functional composition and diversity, balanced Hill-number analyses, and exploratory patch-attribute associations

Table S1. Herbaceous plant checklist and species origin

Scientific name	Origin	No. plots	No. patches
Achyranthes aspera L.	Native	46	14
Acrostichum aureum L.	Native	1	1
Adiantum caudatum L.	Native	3	3
Alocasia odora (Roxb.) K. Koch	Native	105	26
Alpinia *katsumadai* Hayata	Native	36	12
Alpinia zerumbet (Pers.) Burtt. et Smith	Native	5	2
Anisomeles indica (L.) Kuntze	Native	2	2
Artemisia annua L.	Native	3	2
Arthraxon prionodes (Steud.) Dandy	Native	36	13
Asparagus cochinchinensis (Lour.) Merr.	Native	7	5
Asystasia nemorum Nees	Native	4	3
Bambusa sinospinosa McClure	Native	13	6
Brainea insignis (Hook.) J. Sm.	Native	2	1
Canavalia rosea (Sw.) DC.	Native	2	1
Cardiospermum halicacabum L.	Native	26	9
Cassytha filiformis L.	Native	1	1
Cayratia japonica (Thunb.) Gagnep.	Native	27	10
Celosia argentea L.	Native	1	1
Centella asiatica (L.) Urban	Native	2	1
Cissus pteroclada Hayata	Native	2	1
Cleome viscosa L.	Native	1	1
Coccinia grandis (L.) Voigt	Native	7	2
Colocasia antiquorum Schott	Native	3	1
Crinum asiaticum var. sinicum (Roxb. ex Herb.) Baker	Native	3	2
Cyathula prostrata (L.) Blume.	Native	1	1
Cyclosorus acuminatus (Houtt.) Nakai	Native	1	1
Cyclosorus parasiticus (L.) Farwell	Native	5	2
Cynodon dactylon (L.) Pers.	Native	4	2
Cyperus rotundus L.	Native	2	1
Desmodium triflorum (L.) DC.	Native	5	2
Dicranopteris pedata (Houtt.) Nakaike	Native	1	1
Digitaria sanguinalis (L.) Scop.	Native	3	2
Dioscorea bulbifera L.	Native	2	1
Drymaria cordata (L.) Willdenow ex Schultes	Native	3	1
Dunbaria truncata (Miq.) Maesen	Native	3	1

Scientific name	Origin	No. plots	No. patches
Eclipta prostrata (L.) L.	Native	3	1
Elephantopus scaber L.	Native	2	1
Eleusine indica (L.) Gaertn.	Native	2	1
Epaltes australis Less.	Native	5	2
Epipremnum pinnatum (L.) Engl.	Native	4	2
Euphorbia hirta L.	Native	2	1
Euphorbia humifusa Willd. ex Schltdl.	Native	3	1
Heliotropium indicum L.	Native	3	1
Hydrocotyle sibthorpioides Lam.	Native	4	2
Ipomoea aquatica Forsk.	Native	3	1
Ipomoea obscura (L.) Ker-Gawl.	Native	14	6
Leonurus japonicus Houtt.	Native	2	1
Lepidagathis incurva Buch.-Ham. ex D. Don	Native	1	1
Liriope spicata (Thunb.) Lour.	Native	1	1
Lygodium flexuosum (L.) Sw.	Native	2	1
Lygodium japonicum (Thunb.) Sw.	Native	11	4
Lygodium microphyllum (Cav.) R. Br.	Native	1	1
Merremia vitifolia (Burm. f.) Hall. f.	Native	57	14
Musa basjoo Siebold & Zucc. ex Iinuma	Native	6	3
Nephrolepis biserrata (Sw.) Schott	Native	5	3
Nephrolepis cordifolia (L.) Trimen	Native	7	3
Oenanthe javanica (Bl.) DC.	Native	3	1
Oxalis corniculata L.	Native	6	2
Paederia foetida L.	Native	19	9
Panicum repens L.	Native	2	1
Phyla nodiflora (L.) Greene	Native	1	1
Physalis angulata L.	Native	3	2
Piper sarmentosum Roxb.	Native	67	16
Polygonum chinense L.	Native	17	7
Portulaca oleracea L.	Native	1	1
Pothos repens (Lour.) Druce	Native	2	1
Pteris ensiformis Burm.	Native	2	1
Pteris multifida Poir.	Native	2	1
Pteris semipinnata L.	Native	2	1
Pteris vittata L.	Native	4	1
Pueraria montana (Lour.) Merr.	Native	6	3
Pueraria montana var. lobata (Willd.) Maesen & S.M.Almeida ex Sanjappa & Predeep	Native	10	4
Ranunculus japonicus (Thunb.) Hultén	Native	3	2
Saccharum arundinaceum Retz.	Native	80	23

Scientific name	Origin	No. plots	No. patches
Salvia plebeia R. Br.	Native	3	1
Scoparia dulcis L.	Native	2	1
Senna tora (L.) Roxb.	Native	11	5
Solanum americanum Mill.	Native	10	5
Solanum nigrum L.	Native	18	6
Spermacoce pusilla Wall.	Native	4	2
Sphagneticola calendulacea (L.) Pruski	Native	1	1
Stemona angusta I.R.H.Telford	Native	4	2
Stemona parviflora C. H. Wright	Native	14	6
Stephania longa Lour.	Native	11	5
Talinum paniculatum (Jacq.) Gaertn.	Native	2	1
Thunbergia grandiflora (Rottl. ex Willd.) Roxb.	Native	19	10
Vernonia cinerea (L.) Less.	Native	10	6
Wollastonia biflora (L.) DC.	Native	3	2
Zehneria japonica (Thunb.) H. Y. Liu	Native	10	5
Zoysia japonica Steud.	Native	1	1
Zoysia pacifica (Goudswaard) M. Hotta & S. Kuroki	Native	1	1
Asystasia gangetica (L.) T. Anders.	Alien non-invasive	10	5
Asystasia gangetica subsp. micrantha (Nees) Ensermu	Alien non-invasive	1	1
Atractylodes macrocephala Koidz.	Alien non-invasive	1	1
Bambusa vulgaris f. vittata (Riviere & C. Riviere) T. P. Yi	Alien non-invasive	7	3
Cullen corylifolium (L.) Medik.	Alien non-invasive	1	1
Lavandula angustifolia Mill.	Alien non-invasive	4	1
Maranta arundinacea L.	Alien non-invasive	5	3
Ocimum tenuiflorum Burm. f.	Alien non-invasive	2	1
Spermacoce exilis (L. C. Rich.) DC.	Alien non-invasive	2	2
Syngonium podophyllum Schott	Alien non-invasive	2	2
Tradescantia zebrina Bosse	Alien non-invasive	1	1
Ageratum conyzoides L.	Invasive	1	1
Amaranthus palmeri S. Watson	Invasive	1	1
Amaranthus spinosus L.	Invasive	6	5
Axonopus compressus (Sw.) Beauv.	Invasive	5	4
Bidens alba (L.) DC.	Invasive	62	26
Bryophyllum pinnatum (L. f.) Oken	Invasive	7	4
Chromolaena odorata (L.) R. M. King & H. Rob.	Invasive	97	27
Cyperus involucratus Rottb.	Invasive	2	1
Dysphania ambrosioides (L.) Mosyakin et Clemants	Invasive	1	1

Scientific name	Origin	No. plots	No. patches
Erigeron annuus (L.) Pers.	Invasive	1	1
Erigeron canadensis L.	Invasive	2	2
Ipomoea cairica (L.) Sweet	Invasive	6	5
Malvastrum coromandelianum (L.) Garcke	Invasive	3	2
Melinis repens (Willd.) Zizka	Invasive	1	1
Mikania micrantha Kunth	Invasive	78	27
Mimosa diplotricha C. Wright	Invasive	3	3
Mimosa pudica L.	Invasive	26	14
Panicum maximum Jacq.	Invasive	2	1
Parthenium hysterophorus L.	Invasive	8	6
Passiflora foetida L.	Invasive	16	11
Spermacoce alata Aubl.	Invasive	3	3
Sphagneticola trilobata (L.) Pruski	Invasive	18	10
Stachytarpheta jamaicensis (L.) Vahl	Invasive	6	4
Tridax procumbens L.	Invasive	1	1

Note. Origin categories are Native, Alien non-invasive, and Invasive. No. plots and No. patches denote the number of sampled plots and forest patches in which each herbaceous species was recorded.

Table S2. Mixed-effects model results for taxonomic diversity, herbaceous species origin, and invasive-herb dominance

Panel A. Taxonomic diversity across vegetation layers

Vegetation layer	Response	Model (random structure)	Interior descriptive mean	Edge descriptive mean	Edge − Interior	95% CI	FDR-adjusted *P*
Adult trees	Species richness	COM-Poisson (random intercept)	4.964	4.089	-0.845	-1.283 to -0.407	<0.001
Adult trees	Shannon diversity	Gaussian (random slope)	1.186	1.014	-0.171	-0.361 to 0.018	0.081
Saplings	Species richness	Zero-inflated Poisson (random intercept)	3.964	2.795	-0.378	-0.734 to -0.023	0.0495
Saplings	Shannon diversity	Gaussian (random slope)	0.926	0.710	-0.216	-0.381 to -0.051	0.024
Shrubs	Species richness	Poisson (random intercept)	4.381	3.634	-0.574	-1.045 to -0.103	0.027
Shrubs	Shannon diversity	Gaussian (random slope)	0.958	0.798	-0.160	-0.342 to 0.021	0.081
Herbs	Species richness	Poisson (random intercept)	5.262	7.232	1.936	1.236 to 2.637	<0.001
Herbs	Shannon diversity	Gaussian (random slope)	1.153	1.555	0.402	0.220 to 0.584	<0.001

Panel B. Targeted sensitivity analyses

Response	Analysis	Effect measure	Estimate	95% CI	FDR-adjusted *P*	n patches
Shrub richness	Poisson GEE: exchangeable	Edge/Interior rate ratio	0.829	0.693 to 0.993	0.041	28
Shrub richness	Poisson GLMM: random slope	Edge/Interior rate ratio	0.801	0.637 to 1.007	0.058	28
Shrub richness	Patch-level paired t-test	Edge - Interior species per plot	-0.747	−1.542 to 0.048	0.064	28
Sapling Shannon	Gaussian LMM: all plots, empty = 0	Edge - Interior Shannon	-0.216	-0.381 to -0.051	0.012	28

Response	Analysis	Effect measure	Estimate	95% CI	FDR-adjusted *P*	n patches
Sapling Shannon	Gaussian LMM: non-empty plots only	Edge - Interior Shannon	-0.202	-0.410 to 0.006	0.057	28
Sapling presence	Binomial GEE: sapling occurrence	Edge/Interior odds ratio of sapling presence	0.679	0.449 to 1.026	0.066	28
Native-herb richness	Targeted three-group origin sensitivity (GLMM)	Edge − Interior species per plot	−0.404	−0.897 to 0.090	0.109	28
Alien non-invasive-herb richness	Targeted three-group origin sensitivity (GLMM)	Edge − Interior species per plot	0.050	−0.018 to 0.118	0.151	28

Panel C. Herbaceous species-origin components and invasive-herb dominance

Response	Model (random structure)	Scale	Estimate	95% CI	Raw *P*	FDR *P*
All-herb richness	Poisson (random intercept)	species plot^{-1}	1.936	1.236 to 2.637	<0.001	—
Richness of pooled non-invasive herbs	COM-Poisson (random intercept)	species plot^{-1}	−0.312	−0.824 to 0.199	0.231	0.231
Invasive-herb richness	Poisson (random intercept)	species plot^{-1}	2.256	1.886 to 2.626	<0.001	<0.001
Absolute invasive-herb projected area	Tweedie, log link (random intercept)	m^2 per plot	57.872	44.802 to 70.943	<0.001	<0.001
Relative invasive-herb projected-area share	Ordered beta, logit link (random intercept)	percentage points	36.857	31.275 to 42.438	<0.001	<0.001

Note. Interior and edge values are descriptive plot-level means; Edge − Interior is the final mixed-effects-model contrast. Positive values indicate higher values at edges. The Benjamini–Hochberg adjustment was applied within each prespecified response family.

Table S3. Community-weighted mean traits, trait coverage, and richness-standardized functional diversity

Panel A. Projected-area-weighted community-weighted means (CWMs)

Community subset	Trait	n plots	Interior CWM (z)	Edge CWM (z)	Edge − Interior (z)	95% CI	FDR-adjusted *P*
All herbs	Plant height	196	0.552	0.317	-0.234	-0.409 to -0.059	0.016
All herbs	Specific leaf area (SLA)	196	-0.155	0.257	0.412	0.255 to 0.569	<0.001
All herbs	Leaf area	196	0.837	0.655	-0.182	-0.324 to -0.040	0.016
All herbs	Leaf dry matter content (LDMC)	196	0.280	0.168	-0.112	-0.242 to 0.018	0.091
All herbs	Thousand-seed mass	196	0.449	-0.010	-0.459	-0.577 to -0.341	<0.001
Pooled non-invasive herbs	Plant height	193	0.558	0.577	0.019	-0.136 to 0.173	0.809
Pooled non-invasive herbs	Specific leaf area (SLA)	193	-0.250	-0.294	-0.044	-0.186 to 0.099	0.809
Pooled non-invasive herbs	Leaf area	193	0.886	1.006	0.120	-0.050 to 0.291	0.809
Pooled non-invasive herbs	Leaf dry matter content (LDMC)	193	0.292	0.330	0.038	-0.112 to 0.188	0.809
Pooled non-invasive herbs	Thousand-seed mass	193	0.519	0.499	-0.020	-0.148 to 0.108	0.809

Note. Traits were transformed at the species level where appropriate and z-standardized across herbaceous species with available values before calculating projected-area-weighted CWMs. The pooled non-invasive sensitivity excludes invasive herbs. Confidence intervals are unadjusted 95% CIs; FDR adjustment was applied to *P* values.

Panel B. Trait coverage and analytical use

Trait	Species with final values	Mean projected-area coverage: Edge	Mean projected-area coverage: Interior	Analytical use

Trait	Species with final values	Mean projected-area coverage: Edge	Mean projected-area coverage: Interior	Analytical use
Maximum plant height	126	100%	100%	CWM and FD
Specific leaf area	126	100%	100%	CWM and FD
Individual leaf area	126	100%	100%	CWM and FD
Leaf dry matter content	126	100%	100%	CWM and FD
Thousand-seed mass	111	98.0%	96.1%	CWM only

Panel C. Richness-standardized functional diversity

Functional-diversity metric	Interior mean SES	Edge mean SES	Edge − Interior (SES)	95% CI	FDR-adjusted *P*
Functional richness (FRic)	0.020	0.135	0.115	-0.224 to 0.455	0.731
Functional dispersion (FDis)	-0.185	-0.075	0.110	-0.287 to 0.507	0.731
Functional evenness (FEve)	-0.010	0.244	0.253	-0.386 to 0.893	0.731
Functional divergence (FDiv)	-0.462	-0.537	-0.075	-0.643 to 0.493	0.789
Rao's quadratic entropy (RaoQ)	-0.126	-0.032	0.094	-0.254 to 0.441	0.731

Note. SES, standardized effect size. Functional-diversity indices used $\log_{10}$ maximum plant height, SLA, $\log_{10}$ individual leaf area, and LDMC for 126 herbaceous species. For each patch-position community, species richness and the observed positive projected-area vector were held constant, and species identities were randomly drawn from the study-wide herb species pool 999 times. Edge − Interior estimates are mixed-effects-model contrasts based on all estimable patch-position communities; no metric remained significant after FDR adjustment.

Table S4. Balanced Hill-number analyses across vegetation layers and spatial scales

Panel A. Paired patch-level comparisons of mean plot-level alpha Hill diversity across vegetation layers.

Vegetation layer	Hill order	Edge mean	Interior mean	Edge − Interior	95% bootstrap interval	FDR-adjusted paired *P*
Adult trees	q = 0	4.089	4.964	-0.875	-1.604 to -0.125	0.068
Adult trees	q = 1	3.069	3.617	-0.548	-1.123 to -0.004	0.131
Adult trees	q = 2	2.639	3.070	-0.432	-0.942 to 0.067	0.161
Saplings	q = 0	2.795	3.964	-1.170	-2.021 to -0.390	0.034
Saplings	q = 1	2.357	3.232	-0.875	-1.540 to -0.230	0.041
Saplings	q = 2	2.106	2.788	-0.683	-1.229 to -0.159	0.055
Shrubs	q = 0	3.634	4.381	-0.747	-1.491 to -0.006	0.122
Shrubs	q = 1	2.739	3.217	-0.478	-1.017 to 0.080	0.161
Shrubs	q = 2	2.330	2.730	-0.400	-0.871 to 0.056	0.161
Herbs	q = 0	7.232	5.262	1.970	1.006 to 2.818	0.002
Herbs	q = 1	5.099	3.513	1.586	0.956 to 2.159	<0.001
Herbs	q = 2	4.191	2.895	1.296	0.792 to 1.782	<0.001

Panel B. Herbaceous Hill alpha, study-wide pooled gamma, and multiplicative beta diversity under balanced study-wide pooled selection

Hill order	Component	Balanced edge mean	Interior value	Edge − Interior	2.5%–97.5% selection range	n selections
q = 0	Alpha	7.231	5.262	1.969	1.667 to 2.262	4999
q = 0	Gamma	106.012	88.000	18.012	10.000 to 25.000	4999
q = 0	Beta	14.661	16.724	-2.063	-2.993 to -1.243	4999
q = 1	Alpha	4.735	3.168	1.567	1.379 to 1.772	4999

Hill order	Component	Balanced edge mean	Interior value	Edge − Interior	2.5%–97.5% selection range	n selections
q = 1	Gamma	23.782	27.469	-3.687	-5.540 to -1.806	4999
q = 1	Beta	5.024	8.671	-3.647	-4.013 to -3.279	4999
q = 2	Alpha	3.554	2.348	1.206	1.048 to 1.410	4999
q = 2	Gamma	13.063	15.504	-2.441	-3.353 to -1.417	4999
q = 2	Beta	3.677	6.603	-2.927	-3.214 to -2.627	4999

Note. Hill q = 0 is species richness; q = 1 is the exponential of Shannon entropy; and q = 2 is the inverse Simpson concentration. To balance sampling intensity, each selection retained three of four edge plots in every patch (84 edge plots) and compared them with the three interior plots per patch (84 interior plots). Panel A reports the 28 paired patch comparisons; Panel B reports sensitivity to 4,999 balanced study-wide pooled selections. The selection ranges in Panel B are not confidence intervals for patch sampling.

Table S5. Exploratory associations between patch attributes and balanced herb-layer edge effects

Response	Patch attribute	n	Spearman *ρ*	Raw *P*	FDR-adjusted *P*
Herb Hill alpha (q = 0)	Core area index at 50 m (CAI50)	28	0.257	0.187	0.811
Herb Hill alpha (q = 0)	Log isolation distance (NBAR_DIST + 1)	28	-0.094	0.634	0.811
Herb Hill alpha (q = 0)	Log patch area (ha)	28	0.055	0.781	0.852
Herb Hill alpha (q = 1)	Core area index at 50 m (CAI50)	28	0.184	0.347	0.811
Herb Hill alpha (q = 1)	Log isolation distance (NBAR_DIST + 1)	28	-0.102	0.606	0.811
Herb Hill alpha (q = 1)	Log patch area (ha)	28	0.083	0.676	0.811
Herb Hill alpha (q = 2)	Core area index at 50 m (CAI50)	28	0.106	0.591	0.811
Herb Hill alpha (q = 2)	Log isolation distance (NBAR_DIST + 1)	28	-0.098	0.620	0.811
Herb Hill alpha (q = 2)	Log patch area (ha)	28	0.136	0.491	0.811
Invasive-herb relative projected-area share	Core area index at 50 m (CAI50)	28	0.022	0.912	0.912
Invasive-herb relative projected-area share	Log isolation distance (NBAR_DIST + 1)	28	0.235	0.228	0.811
Invasive-herb relative projected-area share	Log patch area (ha)	28	-0.378	0.048	0.571

Note. Balanced edge effects were calculated as Edge − Interior values at the patch level. Patch area and nearest-neighbour distance were log_{10}-transformed; CAI50 is the core-area index at a 50-m edge depth. Benjamini–Hochberg FDR adjustment was applied across the 12 prespecified association tests. None of these exploratory associations was supported after FDR adjustment.

Figure S1. Plot-level Hill diversity across vegetation layers. Points show mean plot-level Hill diversity (effective species) and bars show 95% confidence intervals for edge and interior plots. q = 0, q = 1, and q = 2 represent species richness, the exponential of Shannon entropy, and the inverse Simpson concentration, respectively.

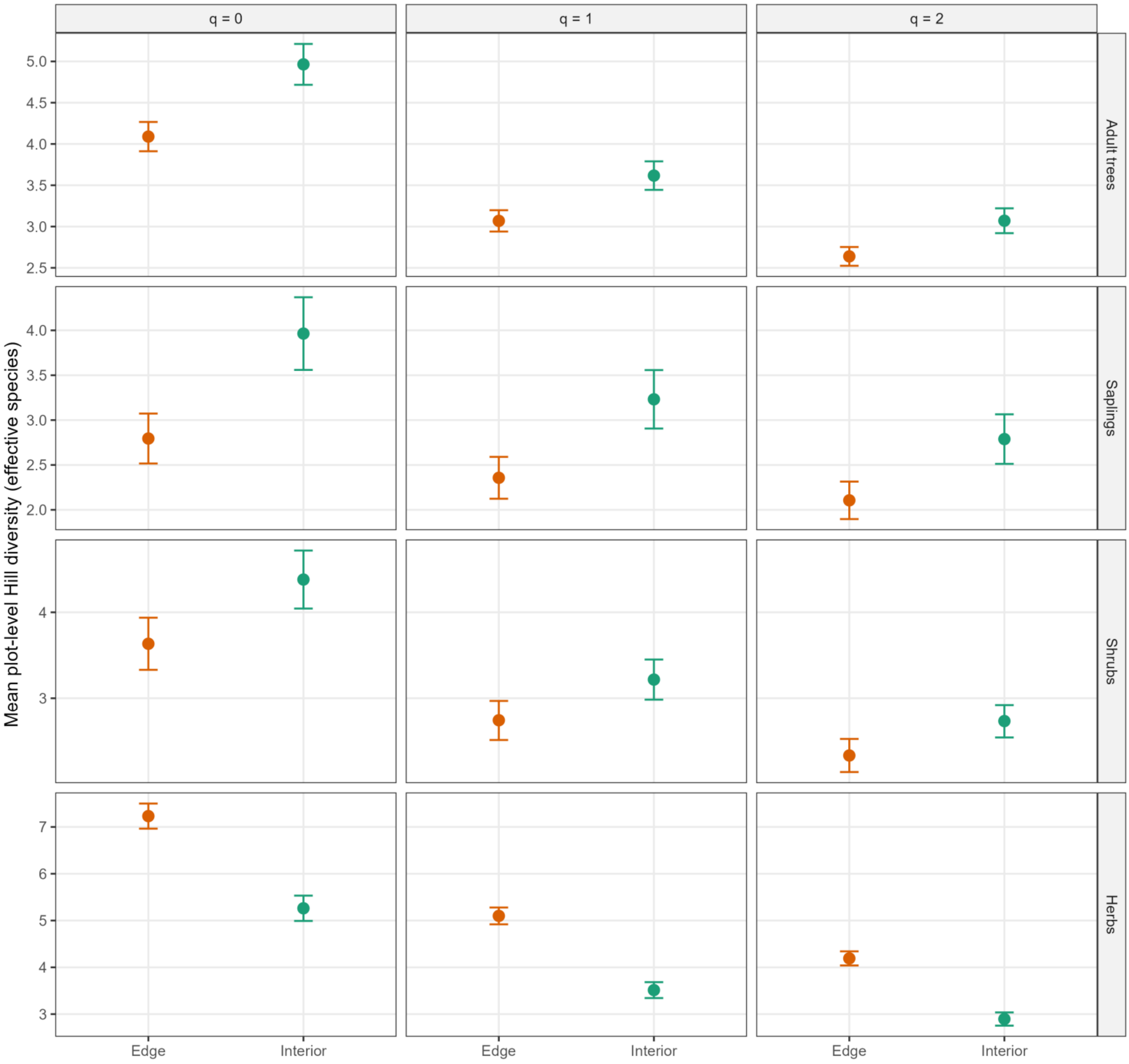

Figure S2. Balanced patch-level Hill-diversity effects across vegetation layers. Points show paired mean differences (Edge − Interior) and horizontal bars show 95% bootstrap intervals across 28 forest patches. The upper row reports mean plot alpha diversity; the lower row reports patch-position gamma diversity. The dashed vertical line denotes no difference.

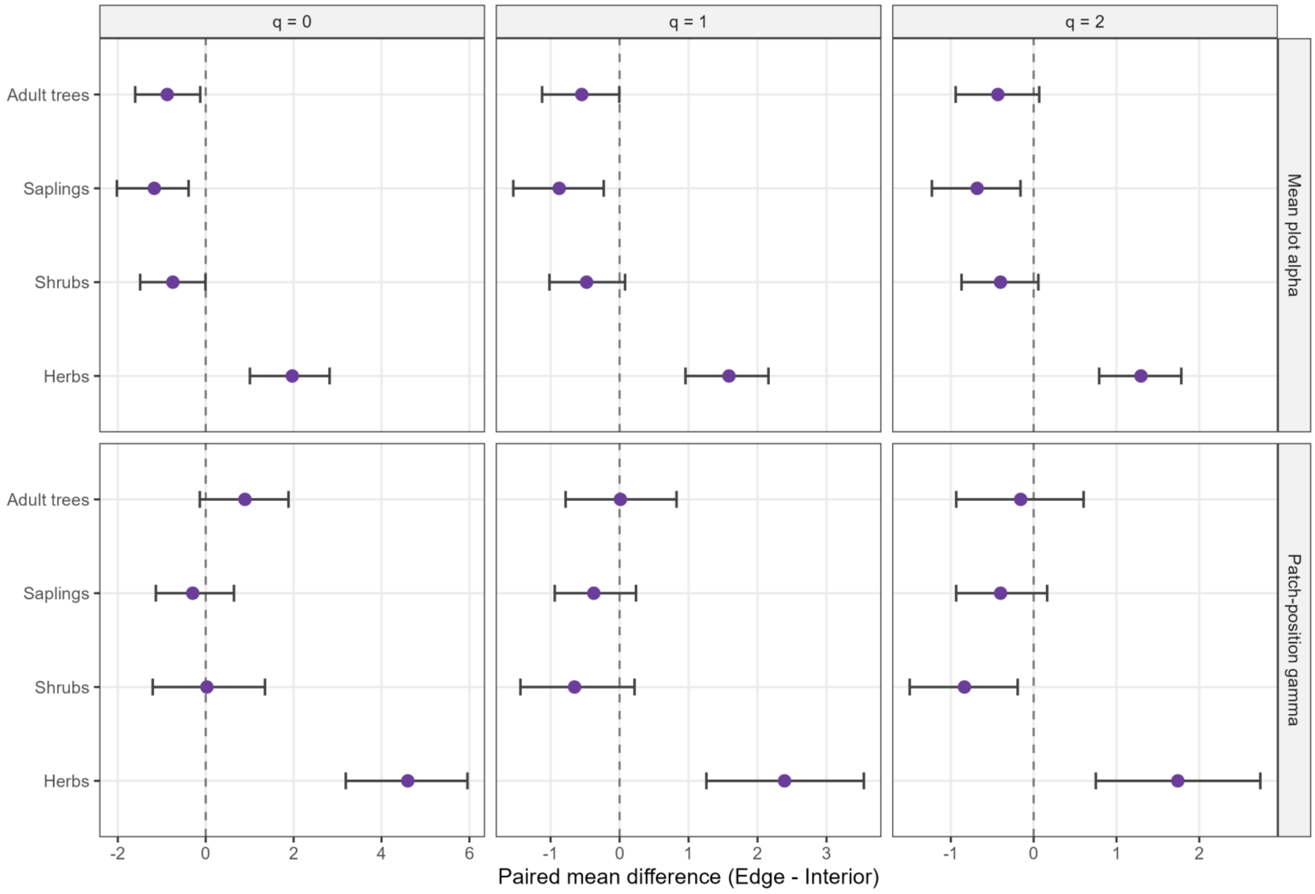

Figure S3. Sensitivity of among-patch compositional differentiation to the exclusion of widespread invasive herbs. The horizontal axis shows mean edge–edge dissimilarity minus mean interior–interior dissimilarity for the full herbaceous community and after excluding the three or five invasive herb species with the highest frequencies across all plots. Negative values indicate lower dissimilarity among edge communities. Points show paired bootstrap means and horizontal bars show 95% bootstrap intervals.

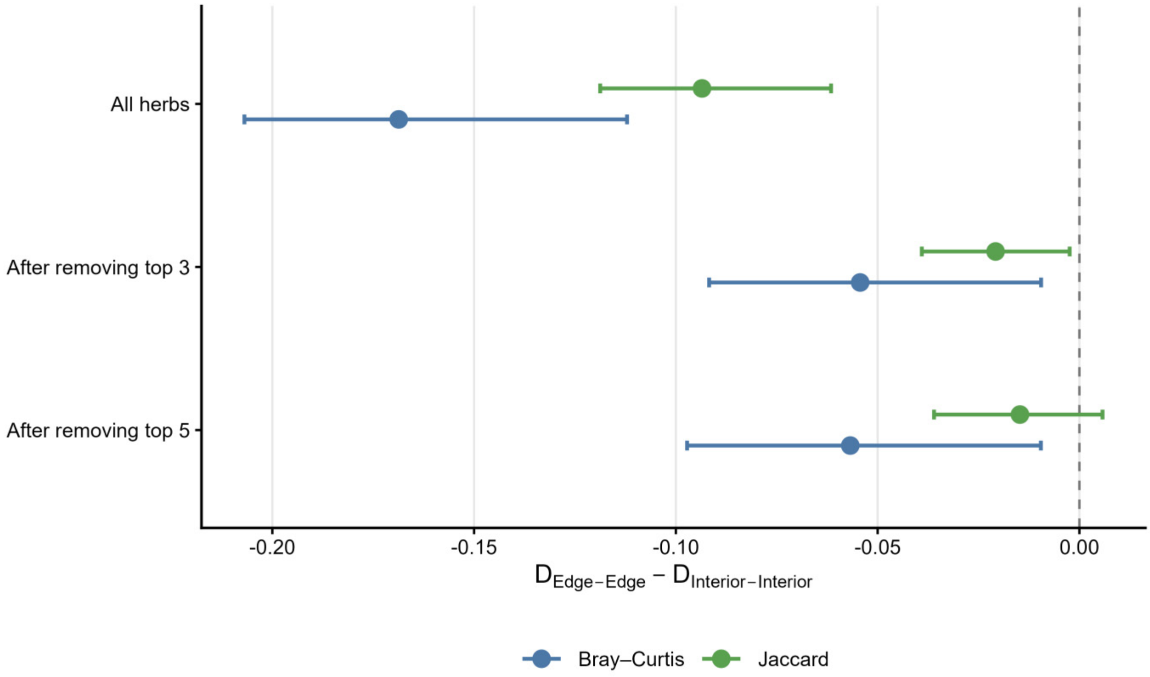

Dataset S1 (separate Excel workbook). Quantitative vegetation records, plot and patch metadata, species-origin metadata, and layer-specific quantitative community matrices for the 196 plots in 28 forest patches.
